# mRNA-based therapeutics in lung Cancer: Mechanisms, applications, and translational challenges

**DOI:** 10.1016/j.jgeb.2026.100717

**Published:** 2026-06-05

**Authors:** Iman Wehbe, Abdelaziz Tlili

**Affiliations:** a Department of Applied Biology, College of Sciences, University of Sharjah, P.O. Box 27272, Sharjah, United Arab Emirates; b Research Institute of Science and Engineering, University of Sharjah, P.O. Box 27272, Sharjah, United Arab Emirates

**Keywords:** Non-small cell lung cancer (NSCLC), Small cell lung Cancer (SCLC), mRNA-based therapeutics, Lipid nanoparticles (LNPs), Tumor microenvironment, Gene editing, Targeted therapy, Immunotherapy

## Abstract

Lung cancer remains one of the most prevalent and lethal malignancies worldwide owing to late diagnosis, molecular heterogeneity, and development of therapeutic resistance. Recent global cancer statistics indicate that it accounts for approximately 2.5 million new cases and 1.8 million deaths annually. The rise of messenger RNA (mRNA) technology has become a highly effective approach in the fight against infectious diseases, particularly highlighted by its successful use in COVID-19 vaccines. Accordingly, mRNA-based therapeutics have emerged as a versatile and adaptable approach with a high potential to address the limitations of conventional cancer treatments. This review aims to provide a comprehensive overview of the available cancer treatments and their limitations, summarize the molecular and cellular mechanisms involved in lung cancer progression, and introduce the mRNA-based approach and its application in lung cancer, including mRNA vaccines, targeted protein therapy, immune-modulating therapy, gene editing, and cellular reprogramming approaches. Furthermore, we discuss the main mechanisms underlying mRNA-based therapies, address current applications, and evaluate approaches to overcome the limitations and challenges associated with mRNA-based therapies. Overall, mRNA-based therapy represents a flexible and rapidly evolving approach that, with further research and development, will reshape precision treatment of lung cancer.

## Highlights

• Comprehensive overview of the available cancer treatments and their limitations.
• Introduce the mRNA-based approach and its application in lung cancer.
• Address limitations and gaps associated with mRNA-based therapies.
• Recourse for researchers in RNA therapies and oncology.

## 1 Introduction

Lung cancer (LC) remains one of the most prevalent and lethal malignancies worldwide. It holds the distinction of being both the most frequently diagnosed cancer and the leading cause of cancer-related deaths.^1^, ^2^ According to recent data from the Cancer today, International Agency of Research on Cancer (IARC), approximately 2.5 million new cases and 1.8 million deaths are reported annually, highlighting the persistent and growing global burden of this disease.^3^ LC incidence and mortality rates vary between geographical areas due to factors such as industrial pollution, smoking prevalence, and healthcare accessibility and limitations.^4^, ^5^, ^6^ The risk of LC is increased by environmental factors, such as exposure to air pollution, asbestos, biomass fuels, and radon, among other carcinogens, with tobacco smoke remaining the leading cause.^5^, ^6^, ^7^ Despite advances in treatment, LC survival rates are still low, which emphasises the continued need for better therapies and public health campaigns.^4^, ^8^ In some cases, patients with LC have some of the lowest 5-year survival rates.^9^

According to histopathological and molecular analysis, lung cancer is a heterogeneous disorder that arises through complex multistep processes characterised by various genetic and epigenetic alterations.^10^ These changes include the activation of pathways that promote cellular growth and the suppression of tumor suppressor pathways.^11^ The two primary forms of lung cancer are non-small cell lung cancer (NSCLC), which includes subtypes such as adenocarcinoma, squamous cell carcinoma, and large cell carcinoma, and small cell lung cancer (SCLC). NSCLC is the more prevalent form, accounting for 80–85% of all cases, while the remaining 15–20% correspond to SCLC.^2^, ^5^, ^8^, ^12^

NSCLC accounts for the majority of cases and is characterised by a heterogeneous spectrum of actionable genetic alterations, including EGFR mutations, ALK rearrangements, and KRAS mutations, making it more amenable to targeted therapeutic approaches, including emerging mRNA-based strategies. In contrast, SCLC is a highly aggressive neuroendocrine subtype with rapid progression, early metastasis, and a relatively limited repertoire of actionable oncogenic drivers.^2^, ^5^, ^8^, ^12^ This heterogeneity presents a significant challenge for effective treatments, as tumors often tend to exhibit variable molecular profiles and adaptive resistance mechanisms, which are further elaborated in Section 3.

Current lung cancer treatment is multidisciplinary and incorporates various therapeutic strategies. Choosing the treatment is complex and depends on several factors, including the cancer subtype, disease stage, patient's overall health, age, and, more recently, the patient's molecular profile. Available treatment options include surgery, chemotherapy, radiotherapy, targeted molecular therapy, immunotherapy, and palliative care.^12^ While these modalities have improved patient outcomes in specific cases, their long-term effectiveness remains limited due to several factors, such as treatment toxicity, selectivity, the development of drug resistance, tumor heterogeneity, and restricted effectiveness in advanced and metastatic cases.^13^, ^14^, ^15^, ^16^, ^17^ Nevertheless, ongoing research focuses on overcoming these challenges by combining existing therapeutic approaches and developing novel treatment strategies to improve the outcomes of patients with LC.

Messenger RNA (mRNA) is a molecule that carries genetic information from DNA to ribosomes to be translated into proteins, depending on the cell type, requirements, and circumstances.^18^ Advances in molecular biology and in vitro transcription technologies have enabled the synthesis of functional mRNA molecules, paving the way for their therapeutic use.^19^ In 1990, a landmark study demonstrated that direct injection of mRNA into mouse skeletal muscle could produce efficient in vivo protein expression, providing the first evidence that mRNA is a potential therapeutic agent.^20^ In oncology, mRNA-based approaches offer several advantages, including rapid design, flexible antigen targeting, and the ability to stimulate strong immune responses. These features make mRNA an attractive platform for cancer vaccines and other therapeutic strategies aimed at enhancing antitumor immunity.^21^, ^22^, ^23^

This review aims to provide a comprehensive overview of the development, mechanisms, and therapeutic potential of mRNA-based treatments for lung cancer. This review focuses on lung cancer biology, mechanisms of mRNA-based therapies, current applications, and strategies to overcome existing limitations.

## 2 Literature search and selection criteria

A comprehensive literature search was conducted using the PubMed, Scopus, and Web of Science databases to identify studies related to lung cancer biology and mRNA-based therapeutics. The search strategy included combinations of the following keywords: “*lung cancer*”, “*non-small cell lung cancer*”, “*molecular basis*”, “*mRNA therapy*”, “*mRNA therapeutics*”, “*mRNA vaccines*”, “*RNA-based therapeutics*”, “*lipid nanoparticles*”, and “*cancer therapy*”. Peer-reviewed articles published in English between 2010 and 2025 were considered, and the main landmark studies were mentioned when necessary to provide historical and conceptual context for mRNA technology. Following the initial screening (*n* = 239 publications), 213 studies were selected based on the relevance of the titles and abstracts. Full-text evaluation was subsequently performed to include studies addressing lung cancer mechanisms, therapeutic limitations, mRNA-based development and delivery systems, and relevant clinical applications.

## 3 Molecular basis of lung cancer

As previously mentioned, lung cancer is classified into NSCLC and SCLC, two categories that differ not only in histopathology and clinical behavior but also in their molecular architecture and therapeutic vulnerabilities. NSCLC is characterised by a heterogeneous spectrum of oncogenic driver mutations, whereas SCLC is associated with a higher tumor mutational burden (TMB), an extremely aggressive molecular profile, rapid relapse, and frequent loss of key tumor suppressor genes. The ability to distinguish between them is important, as they influence the disease progression and shape the therapeutic strategies that will be followed.^2^, ^5^, ^8^, ^24^

Lung cancer arises from a complex interplay of molecular and cellular mechanisms that underlie tumor initiation, progression, metastasis, and resistance to therapy.^25^, ^26^ At the genetic level, mutations in key oncogenes and tumor suppressor genes disrupt cellular balance and promote malignancy.^27^

Several oncogenes are frequently mutated or amplified in lung cancer, including Epidermal Growth Factor Receptor (*EGFR*), *Kirsten Rat Sarcoma Viral Oncogene Homolog* (*KRAS*), *B-Raf Proto-Oncogene, Serine/Threonine Kinase* (*BRAF*), and *Phosphatidylinositol-4,5-Bisphosphate 3-Kinase Catalytic Subunit Alpha* (*PIK3CA*).^26^ These mutations activate growth-promoting signaling pathways, such as EGFR, MAPK, and PI3K/AKT/mTOR. Consequently, these changes lead to increased cell proliferation, enhanced cell survival, and resistance to apoptosis. In contrast, the inactivation or deletion of tumor suppressor genes, including *Tumor Protein p53* (*TP53*), *Retinoblastoma 1* (*RB1*), Phosphatase and Tensin Homolog (*PTEN*), and Liver Kinase B1 (LKB1), impairs the cell's ability to repair DNA damage, creating genomic instability and dysregulating cell cycle progression.^28^ This impairment promotes tumor development and aggressive clinical behavior.^11^, ^26^, ^29^, ^30^, ^31^

Beyond genetic mutations, epigenetic modifications represent inheritable changes in gene expression that occur without altering the DNA sequence.^32^ These modifications encompass aberrant changes in DNA methylation, histone modifications, and dysregulation of non-coding RNAs, particularly microRNAs (miRNAs) and long non-coding RNAs (lncRNAs).^33^, ^34^ Collectively, these epigenetic modifications contribute to tumor cell plasticity, uncontrolled proliferation, metastasis, and resistance to therapy in lung cancer.^30^, ^34^

Another defining characteristic of lung cancer is intratumoral and intertumoral heterogeneity.^35^, ^36^, ^37^, ^38^ At the molecular level, this heterogeneity arises from the coexistence of genetically and phenotypically distinct cell populations within the same tumor. These subpopulations often harbor different driver mutations, exhibit distinct epigenetic landscapes, display diverse transcriptomic profiles, and activate different signaling pathways.^39^ Moreover, heterogeneity creates biological differences between primary tumor lesions and metastatic tumors within the same individual.^38^, ^40^

This molecular feature extends beyond malignant cells and contributes to shaping the tumor microenvironment (TME), which plays a critical role in tumor progression, adaptation, and immune evasion.^41^ These processes are achieved by key stromal components such as cancer-associated fibroblasts (CAFs) and the extracellular matrix (ECM).^42^, ^43^ The CAFs remodel and reinforce the ECM by reorganizing major structural components such as collagen, fibronectin, and hyaluronan, ultimately leading to the formation of a dense, highly cross-linked stromal network^42^, ^43^. Continuous ECM remodeling increases tissue stiffness and the interstitial fluid pressure within the TME rather than the entire lung. As a result, this physical restriction within the tumor architecture reduces vascular perfusion (blood flow) and significantly impairs treatment delivery as well as immune cell activation and proliferation.^42^, ^43^ These stromal barriers, discussed further in Section 7.1, present significant challenges for nanoparticle-based delivery systems, especially lipid nanoparticles (LNPs).

Additionally, epithelial-to-mesenchymal transition (EMT) allows tumor cells to acquire invasive and migratory properties, thereby facilitating metastatic dissemination.^44^ Lung tumors also utilize multiple immune evasion strategies, including reduced antigen presentation, increased expression of immune checkpoint molecules, and recruitment of suppressive immune cells, such as regulatory T cells (Tregs) and myeloid-derived suppressor cells (MDSCs).^45^, ^46^ Moreover, tumor-induced angiogenesis, driven by factors such as vascular endothelial growth factor (VEGF) and fibroblast growth factor-2 (FGF-2), ensures an adequate blood supply for tumor growth and promotes dissemination.^47^, ^48^

Understanding the causes of cancer remains a challenge owing to its complex molecular nature. However, advances in genomics, molecular biology, and biotechnology have substantially improved our understanding of the mechanisms underlying lung cancer development and progression. These advances have helped shed light on the intricate molecular nature and mechanisms of lung cancer, thereby identifying a range of therapeutic targets, including EGFR inhibitors, immune checkpoint blockers, and RNA-based treatments, all aimed at disrupting specific pathways crucial to lung cancer development. These advances are paving the way for more precise and personalized therapeutic approaches tailored to the molecular characteristics of individual tumors.

## 4 Limitations of current therapies

Despite major advances in lung cancer management, currently available therapeutic strategies, including surgery, chemotherapy, radiotherapy, targeted therapy, and immunotherapy, are limited. Although these modalities have contributed to improved survival in selected patients and populations, significant challenges continue to limit their long-term clinical effectiveness.

Although surgery remains the fundamental choice of treatment, especially for patients diagnosed with early-stage lung cancer,^49^, ^50^, ^51^ several challenges limit its applicability and effectiveness. These include high procedural costs, the necessity for a highly skilled and experienced surgeon, and potential postoperative complications such as infections, bleeding, and the formation of blood clots, along with an often-extended recovery period.^52^, ^53^

Chemotherapy involves the use of cytotoxic drugs and chemicals,^54^ which are administered to inhibit tumor growth or suppress cellular proliferation.^55^ Despite its high demand and effectiveness, chemotherapy has significant limitations and risks. These drugs are designed to attack rapidly dividing tumor cells; however, they are non-selective and can also damage rapidly dividing healthy cells, such as those found in the bone marrow, gastrointestinal system, and hair follicles.^13^ Another critical limitation is the development of chemoresistance,^14^ which reduces the effectiveness of the treatment and increases the likelihood of cancer relapse and disease progression.

Radiotherapy treats cancer by exposing tumor tissues to high doses of ionizing radiation.^56^, ^57^ This treatment activates several molecular pathways, initially the DNA damage response (DDR) pathway, leading to cell cycle arrest, apoptosis, or cellular senescence.^58^, ^59^, ^60^ However, a major challenge of radiotherapy is the potential damage to surrounding healthy tissues and organs, which may lead to long-term complications, including cardiovascular diseases and secondary malignancies. Futhermore, radiotherapy can induce chronic inflammation or trigger autoimmune reactions.^61^ Another challenge associated with radiotherapy is the development of radiation resistance in the tumor cells. Cancer cells can adapt by activating various protective mechanisms, including enhanced DNA repair capabilities and the development of hypoxic conditions within the TME. These adaptations help tumor cells evade treatment and reduce the effectiveness of radiation.^15^

Recent advancements in molecular biology, cancer genetics, and diagnostic technologies have revolutionized cancer treatment and enabled the development of more selective therapeutic strategies, namely targeted molecular therapies. Unlike conventional treatments, these therapies are designed to selectively inhibit key molecular alterations in cancer cells, including mutated genes, dysregulated proteins, and aberrant signaling pathways that drive tumor initiation, progression, and survival.^62^ Despite their clinical success, targeted therapies have several important limitations. A major challenge is the development of acquired resistance, which is driven by the adaptive and evolving nature of cancer cells.^63^ In addition, tumor heterogeneity limits their effectiveness, as these therapies are often only applicable to patients harboring specific genetic alterations and biomarkers.^64^ Furthermore, targeted therapies are associated with high costs and require continuous molecular monitoring and advanced diagnostic testing, which may limit their broader clinical implementation.^65^

Immunotherapy refers to a medical treatment that enhances the patient's immune system to recognise and eliminate tumor cells.^66^ Immunotherapy has significantly transformed oncology, providing new opportunities for the development of personalized treatments and extended survival rates, and has shown the potential to achieve durable responses in certain patients.^67^, ^68^ However, despite these advances, immunotherapy is associated with several significant limitations and challenges. Only a subset of patients demonstrate durable responses to immunotherapy due to tumor heterogeneity and immune evasion mechanisms, as discussed in the Molecular Basis Section 3.^16^, ^17^ Many patients develop immune-related adverse events (irAEs), in which immune activation leads to damage to healthy organs and tissues.^15^ Other challenges include high treatment costs, variability in biomarkers, and the capacity of tumor cells to develop resistance, all of which limit the broader clinical applicability of immunotherapeutic strategies.^69^

The major limitations of the current therapeutic approaches for lung cancer are summarized in Table 1.

**Table 1:** Limitations of Current Standard Lung Cancer Therapy.

Therapy Type	Main Limitation(s)	Reason(s) for Limitation(s)
Surgery	Limited to operable early-stage disease, possibility of recurrence.	Lung cancer is often detected at advanced stages, presence of micro-metastases, tumor heterogeneity.^10^, ^52^
Chemotherapy	Chemoresistance, systemic toxicity, non-specific targeting	Activation of DNA repair mechanisms, evasion of apoptosis; increased drug efflux.^13^, ^55^, ^70^
Radiotherapy	Local toxicity, radioresistance.	Damage to surrounding normal tissues, enhanced DNA repair capacity, tumor hypoxia.^56^, ^70^, ^71^, ^72^
Targeted Therapy	Transient-responses acquired resistance.	Secondary mutations, reactivation of signaling pathways.^62^, ^64^, ^65^, ^73^
Immunotherapy	Variability in patient responses, immune evasion.	PD-L1 expression, immunosuppressive TME.^63^, ^66^, ^70^

PD-L1, Programmed Death Ligand-1; TME, Tumor Microenvironment.

## 5 Principle of mRNA therapies

The real potential of mRNA-based therapies is highlighted by the central dogma of molecular biology, which explains how genetic information typically flows from DNA to RNA and ultimately results in protein formation.^74^, ^75^ In addition, mRNA-based therapies provide a highly versatile and programmable platform that enables the precise design of mRNA sequences that encode a wide range of therapeutic proteins, thereby supporting targeted interventions in cancer treatment.^74^, ^75^

mRNA-based therapies for lung cancer utilize carefully designed synthetic mRNA molecules to instruct host cells to produce specific proteins in situ, including antigens, cytokines, or antibodies with antitumor activity. Once delivered into the target cells, the mRNA is translated into the desired therapeutic proteins without being integrated into the host genome. Importantly, mRNA expression is transient because mRNA molecules are naturally degraded by host cells. This transient and non-integrative nature contributes to the safety and controllability of mRNA-based therapeutics, making them an attractive option for clinical applications.^19^, ^76^, ^77^

When designing synthetic mRNA molecules, the goal is to closely mimic endogenous mRNAs while optimizing structural features to enhance stability, translation efficiency, and safety, as well as to minimise innate immune activation.^78^ This process starts by selecting the DNA template that encodes the desired therapeutic protein, antigen, cytokine, or antibody, followed by the incorporation of optimized untranslated regions (UTR's) to reduce secondary structure formation and improve translational efficiency.^78^ This is followed by the incorporation of a functional 5′-cap and a poly-(A) tail to enhance ribosome recruitment and protect the mRNA from degradation. To further improve stability, chemical modifications are made to the nucleosides and ribose sugar, and controlled procedures must be followed to remove any impurities.^78^

However, both endogenous and synthetic mRNA molecules are highly negatively charged, which prevents them from crossing the cell membrane and are susceptible to rapid degradation.^79^, ^80^ Furthermore, mRNA may trigger nonspecific immune activation and is subject to rapid renal clearance.^79^, ^81^ Therefore, specialised delivery systems, mostly non-viral strategies are required as carriers to protect the synthetic mRNA in the extracellular environment and promote effective cellular uptake by target host cells.^16^, ^82^, ^83^, ^84^ Among these, LNPs, polymeric nanoparticles, and vesicle-based nano-carriers, such as exosome-based have become the leading platforms for clinical and experimental mRNA delivery.^85^, ^86^ Further elaboration and explanation are provided in Section 6.

The final product after synthesis is a naked, negatively charged mRNA molecule.^76^ However, despite being fully synthesized, naked mRNA is rapidly degraded and poorly taken up by cells when administered without an appropriate delivery system. To avoid this challenge, current mRNA therapeutics are encapsulated using LNPs, which enable efficient cytosolic delivery into host cells.^22^

LNPs are composed of four main components: an ionisable cationic lipid, a helper phospholipid, cholesterol, and polyethylene glycol (PEG)-lipid.^87^ Beyond the components, the molar ratios of the lipids are critical for the formulation behavior and will strongly influence key properties such as the pKa of the particle, endosomal escape efficiency, and biodistribution.^88^ The amount of ionisable lipid determines the apparent pKa that is responsible for controlling charge activation in the acidic environment of the endosomes and affects the release of the therapeutic mRNA. Moreover, the balance between phospholipids and cholesterol highly contributes to the stability of the membrane and promotes efficient endosomal escape. The PEG-lipid regulates the stability of the particles to promote stable circulation and influences biodistribution and cellular uptake in vivo.^88^

Furthermore, in recent computational approaches, multisampling algorithm-based molecular docking and molecular dynamics simulations have demonstrated the potential of in silico screening to identify and optimize small molecule inhibitors targeting lung cancer – associated kinases, which offers a complementary approach to the LNP-based mRNA therapy for NSCLC.^89^ Specifically, screening of a large drug library against three lung cancer-associated kinases (RSK4, CDK2, and IGF1R) using multisampling docking algorithms (HTVS, SP, XP) followed by MM/GBSA scoring identified 5-nitroindazole as a multitargeted inhibitor.^89^

Following injection, LNPs interact with serum proteins and cell surface receptors and are then internalized via endocytosis. After internalization, LNPs traffic through the endosomal pathway, where endosomal escape enables the release of mRNA into the cytosol.^22^, ^87^ Once released, the therapeutic mRNA is recognised by the host cellular translational machinery and translated into its encoded protein, which is subsequently degraded through normal cellular pathways. 22, 87 The translated product can perform various therapeutic functions, including replacing a deficient or mutated protein, expressing tumor-associated antigen(s) (TAAs), producing cytokines, or delivering genome-editing tools.^90^

A key feature of mRNA-based therapy is its transient expression, as exogenous mRNA functions exclusively in the cytoplasm and is not integrated into the host genome. After cellular uptake, therapeutic mRNA is translated by ribosomes in the host cytosol to produce the encoded protein; however, mRNA molecules are inherently unstable and are rapidly degraded via endogenous cellular mechanisms.^91^, ^92^, ^93^ Intracellular enzymes, such as ribonucleases (RNases), and regulated mRNA decay pathways, including deadenylation, decapping, and exonuclease-mediated degradation, contribute to this instability, thereby limiting the duration of protein expression. Moreover, therapeutic mRNA lacks replication capabilities, which prevents its long-term persistence and expression within host cells.^91^, ^92^, ^93^

Despite the clinical potential and validity of LNPs, emerging alternative platforms are being explored to overcome their inherent liver tropism, which is a notable limitation for lung-targeted therapeutics.^94^ Polymer-based nano-carriers, including poly(β-amino esters) (PBAEs), polyethyleneimine (PEI)-derived systems, and poly(lactic-*co*-glycolic acid) (PLGA)-based platforms, offer significant design flexibility that allows precise control over many parameters such as particle size, surface charge, and degradation rate.^95^, ^96^ These properties influence biological interactions, including cellular uptake, tissue penetration, and intracellular trafficking. In particular, their ability to facilitate endosomal escape and reduce off-target accumulation highlights their potential for improving mRNA delivery to lung tissues. More recently, exosome-derived vesicles have emerged and gained more attention as a delivery system due to their biocompatibility, low immunogenicity, and their natural ability to mediate intercellular communication.^97^, ^98^ Exosomes can be engineered to display targeting ligands, which enable selective lung tumor delivery.^99^ While both platforms remain largely at the preclinical stage, they represent promising avenues to overcome the biodistribution limitations of conventional LNPs and improve therapeutic specificity in lung cancer treatment.

**Fig. 1:**
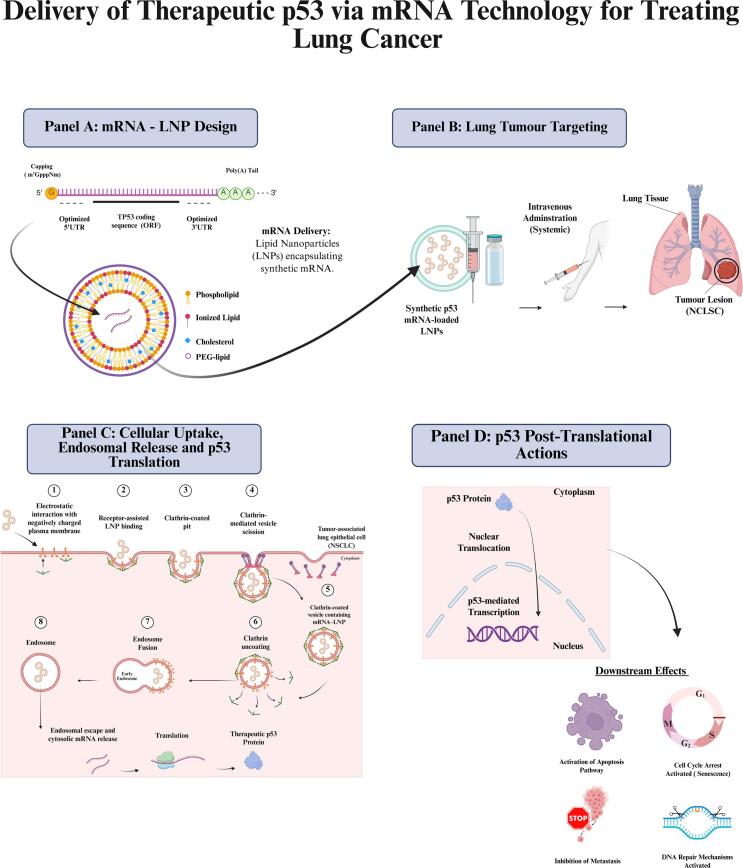
**Systematic diagram representing a synthetic p53 mRNA-based treatment for cancer. Panel A:** Synthetic p53 mRNA is designed and optimized for proper stability and effective translation. Lipid nanoparticles (LNPs) are used to encapsulate the mRNA, which is essential for mRNA protection, and efficient delivery. **Panel B:** Upon injection into a cancer patient, LNPs circulate and preferentially deliver synthetic p53 mRNA to tumor cells. **Panel C:** Cellular internalization occurs via endocytosis, followed by endosomal escape and cytoplasmic release of synthetic mRNA. Recognition by the cellular translation machinery within the cytoplasm, followed by the restoration of p53 expression. **Panel D:** Several downstream pathways are activated once p53 translocates into the nucleus. This therapeutic strategy aims to restore a functional p53 (tumor suppressor protein) in patients.

Table 2 summarizes representative mRNA-based therapeutic candidates currently under investigation or clinical evaluation for lung cancer, including tumor suppressor restoration strategies such as p53 mRNA replacement therapy. To further illustrate the mechanism underlying this strategy, Fig. 1 provides a schematic overview of the design, delivery, and intracellular mechanisms of synthetic p53 mRNA therapy in NSCLC.

**Table 2:** Summary of some of the mRNA drug candidates for lung cancer.

Drug Candidate	Type of mRNA Treatment	Target(s)	Delivery System	Mode of Action	Clinical Trial Phase
BNT116 (BioNTech)	Off-the-shelf mRNA vaccine.	Shared tumor-associated antigens in NSCLC.	LNP-formulated mRNA.	Activates immune T cells against common lung cancer antigens.^115^	Phase I
CV9202† (RNActive®)	mRNA poly-epitope vaccine.	Six NSCLC-associated antigens.	Protamine-formulated mRNA.	Induces immune responses against multiple lung cancer antigens.^116^	Phase I/II (completed)†
mRNA encoding cytokines (IL-12, IL-15, IFN-β)	Therapeutic mRNA.	Immune-stimulatory cytokines.	LNP-based or viral-free delivery.	Enhances anti-tumor immune activity within the TME.^117^, ^118^	Preclinical / early Phase I.
mRNA encoding tumor-suppressor proteins (e.g., p53, PTEN)	mRNA replacement / restoration therapy.	Tumor-suppressor pathways.	LNP-based delivery.	Restores lost tumor-suppressor protein function.^119^	Preclinical.
mRNA for gene editing (CRISPR/Cas9)	Gene-editing mRNA therapy.	Oncogenic driver genes (e.g., KRAS, EGFR).	LNP-based delivery.	Disrupts or corrects cancer-driving genetic alterations.^120^	Preclinical.
mRNA-4157/V940 (Moderna & Merck)	Personalized neoantigen mRNA vaccine.	Patient-specific tumor neoantigens.	LNP-formulated mRNA.	Triggers patient-specific immune responses against tumor mutations.^121^, ^122^	Phase III.
mRNA encoding DLL3-targeting CAR	CAR-T cell reprogramming mRNA.	(*DLL3*) — SCLC-specific surface antigen.	LNP-based or electroporation.	Transiently reprograms T cells to express DLL3-targeting CARs, enabling selective cytotoxicity against SCLC cells overexpressing DLL3.^123^	Preclinical.
mRNA encoding RB1/p53 dual restoration†	mRNA replacement / restoration therapy.	*TP53* and *RB1* tumor suppressor pathways	LNP-based delivery.	Co-delivers p53 and RB1 mRNA to restore dual tumor suppressor function lost in SCLC, inducing cell cycle arrest and apoptosis^124^, ^125^	Preclinical.†

LNP, Lipid Nanoparticle; NSCLC, Non-Small Cell Lung Cancer; TME, Tumor Microenvironment; *DLL3*, Delta-like ligand 3; *TP53,*Tumor Protein p53; *RB1*¸ Retinoblastoma Protein.

Clinical trial phases verified as of April 2025 via ClinicalTrials.gov and developer disclosures.

†CV9202 development was discontinued in 2021 following termination of the CureVac–Boehringer Ingelheim collaboration; completed Phase I/II trial data remain published.

†No specific named candidate currently exists for this approach. The strategy is conceptually supported by preclinical evidence of dual tumor suppressor loss in SCLC and mRNA-mediated p53 restoration studies.

Preclinical denotes candidates currently limited to in vitro or animal model investigation.

As illustrated in Fig. 1 (Panel A), the mRNA construct is engineered to have optimized UTRs, a coding region for the TP53, and a poly-(A) tail. The construct is encapsulated within LNPs composed of ionizable lipids, phospholipids, cholesterol, and PEG-lipids. Following systemic administration (Panel B), the LNPs circulate and accumulate at the tumor site, then they are internalized by cancer cells via endocytosis.

Panel C illustrates the stepwise cellular uptake, internalization, and processing of mRNA-loaded LNPs, this is a critical step that strongly influences the effectiveness of mRNA delivery. Following the electrostatic interaction with the plasma membrane (Step 1), LNPs undergo receptor-assisted binding (Step 2) and clathrin-mediated endocytosis (Steps 3–4),^100^, ^101^ resulting in enclosure within clathrin-coated vesicles (Step 5). After the uncoating (Step 6), LNPs are trafficked through early endosomes (Step 7–8). In the acidic environment of the endosomes, the ionizable lipids within the LNPs become protonated.^102^ This facilitates two escape mechanisms: (i) the “proton sponge effect”, which promotes osmotic swelling to rupture the endosome, (ii) the “lipid mixing/membrane fusion”, where the positively charged ionizable lipids interact with the anionic endosomal membrane phospholipids.^100^, ^103^ Both mechanisms collectively enable the release of the therapeutic mRNA into the cytosol, where it is now accessible for ribosomes for translation.

Beyond the intracellular escape, advanced computational dynamics and molecular simulations have proven valuable in optimizing the encapsulation efficiency and stability of LNP-based nano-carriers therapy for lung cancer.^104^ Such in silico strategies offer a resourceful and rational framework to refine the LNP formulation and design prior to any experimental validation.

As shown in Panel D, upon successful translation of delivered p53 mRNA, the restored protein undergoes nuclear translocation, where it functions as a transcription factor, and initiates several transcriptional programs. At the level of cell cycle regulation, p53 transcriptionally regulates Cyclin-dependent kinase inhibitor 1 A (*CDKN1A)* gene that encodes for p21, which inhibits the cyclin-dependent kinase 2 (CDK2)–cyclin E/A complexes, enforcing G1/S checkpoint arrest, and under some conditions, drives the cells toward irreversible senescence. This prevents uncontrolled cell cycle progression and limits the proliferative expansion of malignant cells.^105^, ^106^

At the same time, p53 activates pro-apoptotic B-cell lymphoma 2 (*BCL2*) family such as *BCL2-associated X protein* (*BAX*), *BCL2 Binding Component 3* (*BBC3*) (protein: PUMA), and *Phorbol-12-Myristate-13-Acetate-Induced Protein 1* (*PMAIP1*) (protein: NOXA), these will promote the permeabilization of the mitochondrial outer membrane.^107^, ^108^ This state triggers the release of cytochrome *c* into the cytosol, and the activation of the apoptotic cascade caspase-9/3, promoting programmed cell death.^109^

In parallel, p53 regulates genomic stability by inducing the expression of DNA repair-associated genes such as *Growth Arrest and DNA Damage 45* (*GADD45*). This activation enhances the recognition of DNA damage and promotes DNA repair via the base excision repair (BER), nucleotide excision repair (NER), and homologous recombination (HR) pathways.^110^, ^111^ Furthermore, in the context of metastasis suppression, p53 functions to repress matrix metalloproteinase (MMPs) expression and downregulates EMT regulators, to limit the invasiveness potential.^112^, ^113^

To complement the idea, a recent study focusing on computational investigations including high-throughput molecular docking and molecular dynamics simulations have demonstrated the polypharmacological nature of p53-associated regulatory networks.^114^ This study revealed that p53 does not operate in isolation but rather functions within an interconnected system of proteins, including EGFR, CDK2, Signal Transducer and Activator of Transcription 3 (STAT3), BCL2, and ATP Binding Cassette Subfamily B Member 1 (ABCB1). This network can influence downstream oncogenic and tumor suppressive pathways. Such computational work offers a valuable complementarity to mRNA-based p53 therapy, because the molecular docking and simulation pipelines can map how restored p53 interacts with its regulatory partners and identify small molecules capable of reinforcing these effects**.**

## 6 mRNA delivery systems in lung cancer

Despite the remarkable advances observed in mRNA therapeutic design and development, their clinical translation remains fundamentally limited by delivery. This section systematically reviews the major classes of mRNA delivery systems that are investigated for their lung cancer applications, and analyzes the key lung-targeted delivery strategies, including inhalation routes, ligand-modified targeted delivery, and strategies for respiratory barrier penetration.

### 6.1 Lipid nanoparticles (LNPs):

LNPs remain the most clinically approved approach, and it is an extensively validated platform used for mRNA delivery.^126^, ^127^ LNPs are composed of four main components: an ionisable cationic lipid (which facilitates the encapsulation of mRNA at a low pH and promotes endosomal escape at physiological pH), a helper phospholipid (which supports bilayer structure), cholesterol (which enhances membrane stability and fusion), and polyethylene glycol (PEG)-lipid (which prevents aggregation and prolongs circulation).^87^, ^128^ In lung cancer, the critical component for organ tropism determination and efficient delivery is the ionisable cationic lipid. For instance, Naidu et al. (2025) showed that structural modification of the linker region between the lipid head-group and hydrophobic tails can alter the biodistribution of the LNPs, ultimately promoting a preferential accumulation in pulmonary tissue.^129^ Similarly, the Selective ORgan Targeting (SORT) strategy enables organ-specific delivery by incorporating supplementary lipids that will modulate LNPs surface charge, thereby redirecting distribution away from the liver toward the lungs.^130^ However, LNP-based systems are still limited by their inherent hepatic tropism following systemic administration, potential for reactogenic inflammatory responses, and challenges related to formulation stability, large-scale manufacturing, and reproducibility.

### 6.2 Polymeric nanoparticles:

Polymeric nanoparticles represent a versatile alternative approach, the system is characterised as a highly modified one, where its properties such as composition, molecular weight, and surface chemistry can be tuned to control the delivery behavior. One of the key systems is the poly-(amine-*co*-ester) (PACE), which has emerged and shown to be an effective scaffold in Suberi et al. (2023) transfection experiments.^131^ The study showed an efficient transfection in both lung epithelial cells and antigen-presenting cells (APCs) after inhalation, without inducing any inflammation that is commonly associated with LNPs delivery in the lung. A key barrier in pulmonary drug delivery is the mucus layer that tends to block nanoparticle penetration; this can be addressed by using a dense PEG coating to enhance diffusion or chitosan-based polymers to prolong airway retention by adhering to the mucus layer.^131^, ^132^ Recent work performed by Li et al. (2023) demonstrated that combinatorial library screening of ionizable lipid–polymer hybrid nanoparticles may help in identifying optimized formulations to improve the efficiency of lung targeted delivery.^133^ Although the hybrid systems of lipid–polymer show promising improvements in lung targeting and gene editing potential, polymeric nanoparticles generally remain less efficient in transfection than LNP-based systems and require extensive optimization.

### 6.3 Exosomes and extracellular vesicles (EVs)

EVs, including exosomes, are endogenous biological nano-carriers that offer advantages for lung targeted delivery. Since they are biocompatible, they exhibit low immunogenicity, can naturally interact with target cells, and demonstrate an efficient membrane fusion, all characteristics that are critical when designing a therapeutic drug for lung delivery.^134^ Recent studies using engineered lung-derived EVs demonstrated that they can successfully deliver therapeutic IL-12 mRNA via inhalation administration. Resulting in tumor regression in lung cancer models and an induction of a systemic anti-tumor response.^77^ A major limitation of this strategy is the inefficient mRNA loading into EVs, which has been addressed using engineering techniques such as RNA-binding domains to promote mRNA packing more efficiently and fusogenic proteins to help release cargo inside cells.^134^ Despite these advances, clinical translation is still limited due to challenges in large-scale production, consistency, and standardization.

### 6.4 Virus-like particles (VLPs):

VLPs are non-infectious protein-based nanostructures, their structural surface architecture mimics a native virion, without containing the replicable genetic material; this nature makes them an attractive platform for cancer immunotherapy applications.^135^ Their pathogen-associated structural patterns (PASPs) facilitate efficient uptake by antigen-presenting cells (APCs) such as dendritic cells and macrophages, thereby enhancing priming of tumor-specific T cell responses within the lung tumor microenvironment.^135^ VLPs are classified into two main types: enveloped VLPs, which have a lipid membrane that facilitates membrane fusion and intracellular delivery, and non-enveloped VLPs, which lack the lipid membrane but have a greater structural stability and resistance to degradation.^135^ VLPs application in vaccine design is effective because they have the ability to display multivalent antigen, meaning they can present multiple copies of tumor antigens that will enhance the activation of both B-cell and T cell. However, their use is limited by issues such as pre-existing immunity against viral scaffolds, manufacturing complexity, and limited cargo capacity compared to LNPs.^135^

Table 3 summarizes the size, advantages, and disadvantages of each system in the context of lung cancer mRNA delivery.

**Table 3:** Comparison of mRNA Delivery Systems for Lung Cancer Applications.

Delivery System	Size Range (nm)	Advantages	Disadvantages	Key References
Lipid Nanoparticles (LNPs)	∼60–200	Clinically validated; high encapsulation efficiency; ionizable lipids enable endosomal escape; scalable manufacturing.	Liver tropism; susceptibility to nebulization-induced degradation; potential inflammatory response in airways.	126, 127, 129, 143
Polymeric Nanoparticles (e.g., PACE, PEG-PLGA)	∼100–300	Tunable biodegradability; PEGylation improves mucus penetration; non-inflammatory profile suitable for inhalation.	Lower transfection efficiency than LNPs; complex optimization required.	131, 133
Exosomes and Extracellular Vesicles (EVs)	∼30–200	Natural lung tropism; low immunogenicity; capable of deep alveolar penetration; stable as dry powder for inhalation.	Difficult to scale; inconsistent loading efficiency; complex characterization.	77, 132, 134
Virus-like Particles (VLPs)	∼20–150	High APC uptake via PASP; multivalent antigen display; strong immunogenicity for lung tumor vaccines.	Potential pre-existing immunity; complex engineering; limited mRNA payload capacity.	^135^

### 6.5 Key Strategies for lung-targeted mRNA delivery:

#### 6.5.1 *Inhalation administration:*

This strategy represents the most direct and selective route for mRNA delivery to lung tumors, avoiding hepatic first-pass metabolism which is associated with systemic administration and enabling high local drug concentrations within the lung TME. Delivery of these therapies includes nebulization of liquid nanoparticle suspensions, intranasal and/or intratracheal instillation, and dry powder inhalation.^77^, ^136^

Tang et al. (2023) demonstrated the dual-targeting potential of inhaled mRNA nanoparticles, designing cationic lipid- and hyaluronic acid-coated particles capable of simultaneously targeting both lung tumor cells (via the CD44 receptor) and tumor-associated macrophages following inhalation,^137^ supporting the principle that inhalation combined with targeted particle design can achieve cell-type specificity within the lung TME.

#### 6.5.2 *Ligand-modified targeted delivery to tumor cells and APCs:*

The majority of recent research has focused on developing nanoparticle systems that enhance targeting specificity toward lung cancer cells and the TME. This is primarily achieved by either adding ligands that bind to specific tumor or immune cell receptors, or by modifying lipid composition (SORT) to redirect nanoparticle distribution toward lung tissue and immune compartments.^132^, ^137^

Several targeting ligands are currently under active investigation, including folate, hyaluronic acid, EGFR-binding ligands, and mannose, which collectively enable preferential delivery to malignant cells or antigen-presenting immune cells within the lung. In parallel, the SORT strategy represents a distinct design approach in which nanoparticle lipid composition is modulated to redirect overall biodistribution toward specific organs such as the lungs, rather than targeting individual cell surface receptors, thereby complementing ligand-based cell-specific delivery strategies.^130^

#### 6.5.3 *Respiratory barrier penetration strategies:*

The respiratory tract presents multiple sequential barriers for mRNA delivery: (1) the mucus gel layer, which traps nanoparticles via electrostatic and hydrophobic interactions with mucin glycoproteins; (2) the pulmonary surfactant layer at the alveolar surface, which can destabilize lipid-based carriers; (3) the mucociliary escalator, which clears mucus-trapped particles toward the pharynx; and (4) alveolar macrophage phagocytosis, which can sequester and degrade nanoparticles before they access tumor cells or epithelial APCs.^77^, ^81^, ^132^

Mucus penetration is primarily enhanced through dense PEG brush coatings, which reduce adhesive interactions with mucin glycoproteins and facilitate nanoparticle diffusion through the mucus mesh, while particle size (<200 nm) and near-neutral surface charge further improve mobility by minimizing steric and electrostatic trapping.^138^, ^139^

Pulmonary surfactant can adsorb onto nanoparticles and alter their physicochemical properties, an effect that can be mitigated through rational lipid composition design, particularly optimization of helper lipids and cholesterol content.^140^ Finally, PEGylation and biomimetic surface modifications, including cell membrane-derived coatings or extracellular vesicle mimetics, reduce recognition by alveolar macrophages, thereby decreasing phagocytic clearance and prolonging nanoparticle residence time to improve access to tumor cells and antigen-presenting cells.^141^, ^142^

## 7 mRNA applications in lung cancer

### 7.1 mRNA vaccines

mRNA vaccines represent one of the most advanced applications of mRNA technology in lung cancer. These vaccines are designed to prime and activate naive T cells, leading to the expansion of tumor-reactive T cells that target tumor-associated antigens (TAAs) or patient-specific neoantigens. Upon delivery, antigen-presenting cells (APCs) translate the mRNA, process the encoded antigens, and present them via major histocompatibility complex (MHC) class I and II molecules, thereby activating cytotoxic CD8^+^ T cells and helper CD4^+^ T cells. This antigen presentation results in tumor-specific cytotoxic T lymphocyte (CTL) expansion that can recognise and eliminate tumor cells.^77^, ^144^, ^145^

A clinical trial evaluated the efficacy, immunogenicity, and safety of a self-adjuvanted mRNA cancer vaccine named CV9202, which was used in combination with local radiotherapy for patients with stage IV NSCLC (*n* = 26). The vaccine encodes six NSCLC-associated antigens (NY-ESO-1, MAGE-C1, MAGE-C2, survivin, 5 T4, and MUC-1), designed to enhance immune recognition of tumor cells. The results showed that only 15.4% of patients experienced grade 3 treatment-related adverse events, while 84% of patients developed antigen-specific immune responses, indicating effective immune activation. Disease stabilisation was also observed in 46.2% of patients.^146^ Despite encouraging immunogenicity findings, clinical efficacy remains variable, highlighting the need for improved delivery strategies and rational therapeutic combinations.

### 7.2 Targeted protein therapy (mRNA encoding therapeutic proteins)

mRNA-based approaches can be used to enable the transient in situ expression of functional therapeutic proteins within tumors or immune cells. In the lung cancer context, this application focuses on delivering immunomodulatory and therapeutic proteins, such as pro-inflammatory cytokines, costimulatory ligands, tumor suppressor proteins, pro-apoptotic factors, and enzymes.^147^

Preclinical studies have demonstrated the potential of this approach to enhance in vivo protein expression, immune activation, and antitumor responses. For example, mRNA encoding interleukin-12 (IL-12) has been shown to act as a potent immunostimulatory agent, promoting CD8^+^ T cell activation and increasing interferon-γ (IFN-γ) production.^148^ These findings indicate that transient, localized IL-12 expression can be achieved, particularly within antigen-presenting cells, thereby enhancing immune-mediated tumor control. However, therapeutic success depends largely on efficient delivery, controlled protein expression, tumor penetration, and is further influenced by variability in patient immune responses.

### 7.3 Gene editing systems and mRNA-based therapy:

Gene editing has emerged as a powerful tool to modify genetic and epigenetic alterations driving lung cancer, aiming to correct or disrupt disease-driving mutations (described in Section 3) and restore antitumor responses. This approach can be achieved by two major strategies: ex vivo and in vivo editing.

One of the most widely used tool in mRNA-based therapeutics is CRISPR-Cas9, due to its precision and transient expression profile.^149^, ^150^, ^151^ It is important to note that the CRISPR-based system requires the delivery of two distinct RNA components: Cas9-encoding mRNA and a separate single guide RNA (sgRNA), together, they reconstitute the functional editing complex in the cytoplasm. Once translated, the Cas9 protein associates with the sgRNA to target specific genomic sequences for editing.^149^, ^150^, ^151^

In one of the earliest trials in humans, patients with NSCLC received CRISPR-Cas9-edited T cells, in which the Programmed Cell Death 1 (*PD-1*) gene was knocked out using CRISPR-Cas9 and a guide RNA (sgRNA). The results showed that the edited T cells demonstrated effective *PD-1* disruption, long-term persistence in peripheral blood, and a favorable safety profile with no dose-limiting toxicities or severe cytokine-release syndrome.^152^ Additionally, preclinical studies have explored the direct mRNA-based delivery of gene-editing systems, such as CRISPR-Cas9 mRNA combined with sgRNA targeting oncogenic drivers (e.g., Cyclin-dependent kinase 4, *CDK4*). These results highlight the capability of selective tumor cell targeting and effective tumor elimination in lung cancer models, including KRAS-mutant models.^153^ Together, these studies demonstrate that mRNA-mediated gene editing is a feasible and controllable approach capable of inducing functional changes without permanent genomic integration. However, clinical translation remains limited by delivery efficiency, safety concerns related to off-target activity, and immune-mediated responses.

Despite these promising findings, a particular immunological barrier in mRNA-mediated CRISPR-Cas9 delivery is the inherent immunogenicity of the Cas9 protein itself, which remains a significant limitation for clinical translation.^154^, ^155^ Following the intracellular translation of the delivered mRNA, the expressed Cas9 protein can be recognised as a foreign antigen and presented via MHC class I molecules, thereby triggering CD8^+^ cytotoxic T responses. This immune activation can result in eliminating therapeutically modified cells before the intended genomic correction or disruption is fully achieved, substantially undermining therapeutic efficacy.^154^, ^155^ Moreover, another challenge is pre-existing humoral and cellular immunity against Cas9 that has been significantly reported in human populations, likely due to prior exposure to bacterial species such as *Staphylococcus aureus* or *Streptococcus pyogenes*, from which the commonly used Cas9 variants originate.^156^ To address these barriers, several approaches are being explored, including transient mRNA-based expression to limit protein exposure, the use of less immunogenic Cas9 variants and developing engineering strategies aimed to humanise or de-immunize Cas9.

### 7.4 Cellular reprogramming:

Cellular reprogramming through mRNA-based treatment uses transient modification of the identity, phenotype, or function of immune or stromal cells, thereby enhancing their ability to recognise and eliminate lung cancer cells.^157^, ^158^ This strategy aims to strengthen antitumor immune responses without inducing permanent genetic alterations. In ex vivo procedures, dendritic cells (DCs) or T cells are isolated and electroporated with mRNA encoding tumor antigens or chimeric antigen receptors (CARs) and then reinfused into the patient. This allows for controlled and transient reprogramming of immune cells.^159^, ^160^, ^161^

In 2019, human T cells were engineered to express CARs targeting mesothelin, a tumor-associated antigen in lung adenocarcinoma and mesothelioma.^162^ The results showed enhanced immune activation, evidenced by increased secretion of key effector cytokines, including IL-2, tumor necrosis factor-α (TNF-α), and interferon-γ (IFN-γ). The results obtained showed that mRNA-engineered immune cells exhibited enhanced cytotoxic activity, improved tumor infiltration, and sustained tumor cell death over time. Despite their promising potential, several challenges remain, including the immunosuppressive TME, antigen heterogeneity, limited persistence of reprogrammed cells, and the need for scalable ex vivo manufacturing processes.

### 7.5 Combination therapies:

Although multiple mRNA-based therapeutic strategies have shown promising preclinical and early clinical outcomes, their efficacy as monotherapies for solid tumors, such as lung cancer, remains limited.

In lung cancer, at the cellular level, the TME is highly enriched with immunosuppressive cells such as tumor-associated macrophages (TAMs), MDSCs, and FOXP3^+^ Tregs, all of which inhibit effective anti-tumor response through the secretion of cytokines such as Transforming Growth Factor-beta (TGF-β), IL-10, and IL-35, driving functional impairment of cytotoxic CD8^+^ T cells, which become exhausted and anergic.^163^, ^164^ At the molecular level, key tumor-intrinsic oncogenic drivers, such as EGFR activation, KRAS mutations and Serine/Threonine Kinase 11 / Liver Kinase B1 (STK11/LKB1) loss, will further reinforce immune evasion through reshaping key immunoregulatory pathways. The activation of EGFR promotes pathways such as JAK/STAT3 and AP-1 that increases the expression of PD-L1 (immune checkpoint molecule) on tumor cells. Then there will be an engagement of PD-L1 with PD-1 receptors on T cells resulting in a functional inhibition of T cell signaling, reduction in cytokine production, and exhaustion of T cells.^163^, ^165^, ^166^ In addition, KRAS mutations will constitutively activate downstream signaling pathways such as MAPK and PI3K pathways that will upregulate the production of pro-inflammatory and immunosuppressive cytokines, such as GM-CSF (granulocyte-macrophage colony-stimulating factor) and IL-8. These will facilitate the recruitment and expansion of MDSCs within the TME, thereby suppressing anti-tumor responses by inhibiting CD8^+^ T cell activation, impairing dendritic cell antigen presentation, and generating reactive oxygen species and immunosuppressive enzymes.^167^, ^168^, ^169^ Lastly, the loss of STK11/LKB1 results in the suppression of STING pathway, which is needed for the activation of the innate immunity, meaning that there is less type 1 interferon, and poor dendritic cell maturation.^170^

This has driven growing interest in rational combination approaches that integrate mRNA therapeutics with established treatment modalities, including chemotherapy, radiotherapy, targeted therapy, and immune checkpoint inhibition.

#### 7.5.1 mRNA-based therapy with immune checkpoint inhibitors (ICIs):

The combination of mRNA-based therapies with immune checkpoint inhibitors (ICIs) represents a rational approach to overcome the limited efficacy of ICIs as monotherapy in lung cancer. ICIs targeting the PD-1/PD-L1 axis aim to restore the function of exhausted T cells; however, their effectiveness largely depends on the presence of preexisting tumor-specific immunity, which is absent in many patients. mRNA-based approaches address this limitation by promoting antigen presentation and priming new CD4^+^ helper T cells and CD8^+^ cytotoxic T cells.^146^, ^171^ This dual mechanism enables both the induction of tumor-specific immunity and the reversal of T cell exhaustion. A clinical trial (NCT03164772) showed that combining the mRNA vaccine CV9202 with checkpoint inhibitors, such as durvalumab or atezolizumab, improved tumor responses and reduced disease progression in patients with lung cancer.^145^, ^172^

#### 7.5.2 mRNA-based therapy with chemotherapy:

The combination of mRNA-based therapies with chemotherapy is supported by the ability of chemotherapy to modulate the TME and enhance immune recognition of cancer cells. In addition to its cytotoxic effects, chemotherapy can induce immunogenic cell death, which increases tumor antigen release and presentation, reduces immunosuppression, and promotes upregulation of MHC class I. These effects create a favorable immunological environment, thereby enhancing the efficacy of mRNA-based therapies in promoting tumor-specific immune responses. An early phase I/IIa study demonstrated the feasibility of this combination, using the mRNA vaccine CV9201 following platinum-based chemotherapy in patients with advanced NSCLC.^173^ The results indicated that the vaccine was well-tolerated and induced antigen-specific immune responses, supporting the potential of this combinatorial approach.

#### 7.5.3 mRNA-based therapy with radiotherapy:

Radiotherapy enhances tumor antigen release, increases the production of pro-inflammatory cytokines, enhances antigen presentation, and facilitates the recruitment of immune cells into the TME. When combined with mRNA-based therapies, these effects can be further amplified, sustaining antitumor responses. A phase Ib study evaluated the combination of the mRNA vaccine CV9201 with hypofractionated radiotherapy, and the results demonstrated tolerability and enhanced antigen-specific immune responses.^116^

#### 7.5.4 mRNA-based therapy with targeted therapy:

The combination of mRNA-based therapies with targeted agents is beneficial because of the ability of these therapies to address both the molecular drivers and the immunological mechanisms responsible for treatment resistance. In other words, combining these two strategies is mechanistically grounded to the bidirectional relationship between the oncogenic signaling and immune evasion within the lung cancer TME. Targeted therapies such as EGFR- Tyrosine kinase inhibitors (TKIs) and KRAS inhibitors suppress oncogenic signaling effectively, but their clinical benefits remain limited. The limitation is explained by the emergence of acquired resistance, which is often accompanied by an immunosuppressive nature of the TME. This resistance-associated TME is characterised by reduced MHC class I antigen presentation machinery, reduced tumor-infiltrating T cells density, and upregulation of PD-L1 and other immune checkpoints ligands, and increased MDSC and Treg infiltration. mRNA therapies overcome these barriers through aiding the immune system to recognise and eliminate resistant tumor cells simultaneously restoring immune activity within the TME. Preclinical studies demonstrated that mRNA encoding resistance-associated neoantigens, including EGFR T790M and KRAS mutations, can induce robust tumor-specific CD8^+^ T cell responses against resistant clones that targeted therapy alone cannot eliminate.^162^ In KRAS-mutant NSCLC, mRNA vaccines targeting KRAS G12C have shown strong immunogenicity, while KRAS inhibitors enhance tumor susceptibility to immune-mediated killing, supporting the rationale for this combinatorial approach.^169^, ^174^

#### 7.5.5 Combinations of mRNA-based therapeutic modalities:

Evidence suggests an interaction when mRNA-based modalities are combined. One strategy involves pairing mRNA cancer vaccines with cytokine-encoding mRNA. For instance, intratumoral administration of mRNA encoding for cytokines such as IL-12, interferon-alpha (IFN-α), Granulocyte-macrophage colony-stimulating factor (GM-CSF), and IL-15 has demonstrated potent antitumor efficacy, followed by systemic antigen-specific T cell expansion, increased granzyme B^+^ T cell infiltration, and immune memory formation.^117^, ^175^ Cytokine-encoding mRNA administration facilitates localized and transient cytokine expression, thereby limiting the toxicities associated with systemic cytokine delivery. This combination maximizes the immunostimulatory activity within the TME.^117^ IL-12 mRNA promotes the conversion of M2 macrophages to M1 macrophages and induces the anti-tumor immunity by the release of C-X-C motif chemokine ligand 9/10 (CXCL9/CXCL10) chemokines that will facilitate the recruitment of C-X-C motif chemokine receptor 3 positive (CXCR3^+^) CD8^+^ T cell into the tumor core.^176^, ^177^, ^178^ IFN-α mRNA will restore the MHC I expression to aid in the recognition of the tumor cells, and the GM-CSF mRNA will differentiate and activate dendritic cells to improve antigen presentation and priming of T cell.^175^

In addition, gene editing tools can be combined with tumor-suppressor encoding mRNA to enhance the antitumor efficiencies and effects.^179^, ^180^ CRISPR/Cas9-mediated oncogene disruption or tumor suppressor restoration leads to significant tumor regression in preclinical studies. LNPs delivery enables targeted editing of oncogenic pathways with reduced off-target effects, and it holds potential for synergy with immunotherapy.^179^, ^180^

#### 7.5.6 Sequence / order of combination therapy:

The clinical therapeutic outcome of the combination strategies is not solely dependent on the agents used but is critically influenced by the timing and precise sequence of administration. This is particularly important given the dynamic nature of the TME, where optimal immune activation depends on appropriately ordered therapeutic interventions. For instance, when integrating mRNA therapeutics with immune checkpoint inhibitors (ICIs), sequential administration in which the mRNA vaccine is given prior to ICs is often more effective. In this case the vaccine first primes and expands tumor specific T cells, which is then followed by a functional enhancement via PD-1/PD-L1 inhibition, this order will ensure a stronger and more sustained antitumor response. In contrast, administering ICIs in the absence of prior T cell priming may result in limited efficacy, as there are insufficient activated T cells for the therapy to act upon.^181^, ^182^

When combined with chemotherapy, if administered before vaccination, it may reduce immunosuppressive cells and enhance antigen presentation, while if the combination was reversed the immune activation may be impaired.^183^, ^184^ Similarly, localized radiotherapy prior to mRNA vaccine administration can act as an immune priming system by inducing immunogenic cell death (ICD) and enhancement of tumor antigen release, therefore improving, and expanding subsequent vaccine-driven-T cell priming.^185^ In contrast, concurrent administration may attenuate vaccine-induced responses, as radiotherapy-mediated lymphodepletion can transiently suppress the circulating immune effector populations required for robust mRNA-encoded antigen recognition.

A similar approach is applied to the integration of mRNA therapies with targeted therapies such as EGFR-TKIs or KRAS inhibitors. The administration of targeted therapy before mRNA vaccination reduced tumor burden and suppressing oncogenic pathways. This will establish a more permissive TME, which facilitates improved antigen presentation and reduces the population of immunosuppressive cells. The permissive TME allows effective priming, expansion, and infiltration of tumor-specific cytotoxic T cells. This will result in aiding the mRNA vaccines to be more durable with clinically meaningful antitumor response.^169^, ^174^

As with the combination approaches described earlier, the sequence of administration of mRNA-based modalities are a critical factor influencing the outcomes. Cytokine mRNA delivered simultaneously with or shortly after vaccination may sustain and help amplify T cell priming, whereas non-specific inflammation may be triggered if adminstrated earlier. Moreover, for the gene-editing and tumor suppressive mRNA approaches, applying CRISPR/Cas9-mediated disruption of oncogenic or immunosuppressive pathways prior to tumor suppressor restoration may enhance therapeutic efficacy by first rendering the TME more receptive to subsequent treatments.^117^, ^186^

Collectively, these considerations underscore the imperative for future clinical trial design to incorporate sequencing as a primary variable, rather than an ancillary parameter, in evaluating mRNA-based combination strategies.

### 7.6 mRNA-based therapeutics in SCLC: distinct considerations:

Although the preceding sections have primarily focused on NSCLC, SCLC represents a biologically and therapeutically distinct entity that warrants separate consideration in the context of mRNA-based therapeutics. SCLC accounts for approximately 15–20% of all lung cancers and is characterised by near-universal loss of the key tumor suppressor genes *TP53* and *RB1*, an extremely aggressive phenotype and a propensity for early metastasis and brain involvement.^187^ SCLC is often described as having a relatively restricted genomic landscape, most commonly defined by near-universal inactivation of *TP53* and *RB1*, with fewer recurrent, actionable oncogenic drivers compared to NSCLC. Despite its high metastatic rate, SCLC exhibits immune evasion features that reduce antigen presentation and create a “cold” TME.^188^

#### 7.6.1 mRNA vaccine approaches and SCLC-specific antigen targets:

In contrast to NSCLC, where shared TAAs such as NY-ESO-1, MAGE-C1/C2, survivin, and MUC-1 have been successfully incorporated into multi-antigen mRNA vaccines (e.g., CV9202), the antigen landscape in SCLC is substantially narrower and less well-characterised. Nevertheless, several SCLC-associated antigens have been identified as potential mRNA vaccine targets, including Delta-like ligand 3 (*DLL3*), which is overexpressed on the surface of SCLC cells and largely absent in normal adult tissues, and ASCL1 (achaete-scute homolog 1), a neuroendocrine transcription factor driving a major SCLC molecular subtype.^123^, ^189^ Neoantigen-based mRNA vaccines such as mRNA-4157/V940 for NSCLC, are being explored in SCLC, but their effectiveness may be lower due to the limited availability of high-quality neoantigens per SCLC patient. As a result, SCLC vaccine strategies may rely more on shared lineage-specific antigens, which are often less immunogenic due to central tolerance.

#### 7.6.2 Delivery considerations and chemosensitivity window:

Initially, SCLC is responsive to chemotherapy, but it has the tendency to relapse. This reduces the tumor burden and renders the TME less suppressive, creating an opportunity for mRNA-based therapies to be administered and produce anti-tumor responses.^190^, ^191^ Real-world data in limited-stage SCLC demonstrate that the addition of immune checkpoint inhibitors to standard chemoradiotherapy improves survival outcomes, with prolonged overall survival and progression-free survival compared to chemotherapy alone. These findings support the clinical relevance of immunotherapy in SCLC and reinforce the presence of a modest but actionable anti-tumor immune response.^191^

## 8 Challenges and barriers

The rapid and successful emergence of mRNA-based therapeutics during the COVID-19 pandemic has shifted the global focus in oncology research toward mRNA-based approaches for cancer treatment, particularly for lung cancer.^19^ Despite encouraging preclinical and early clinical findings, the clinical translation of mRNA-based therapies in lung cancer remains constrained by several biological and technological barriers. This section provides an overview of the key barriers that must be addressed to realise the full therapeutic potential of mRNA-based approaches for lung cancer.

### 8.1 Organ targeting and the unique lung barrier:

The effectiveness and selectivity of mRNA-based therapies depend not only on mRNA design and systemic delivery but also on precise targeting and accumulations within lung tumors or antigen-presenting cells (APCs) in the lungs.^192^, ^193^ Currently, clinically approved LNP-formulated mRNA therapeutics show a strong tendency to accumulate in the liver rather than in lung tissues, which reduces the likelihood of reaching the targeted area after administration and increases the risk of off-target effects and systemic toxicity. Moreover, the lung presents unique anatomical and physiological barriers that further hinder efficient drug delivery. These include the surfactant layer, mucus barriers, narrow airway structures, and the presence of alveolar immune cells, such as macrophages, which clear foreign particles and reduce therapeutic uptake.^194^, ^195^, ^196^

### 8.2 Tumor heterogeneity:

Lung cancer is characterised by significant heterogeneity at both the molecular and spatial levels,^38^, ^40^, ^197^ which poses a major challenge for the design and efficacy of mRNA-based therapies. mRNA-based treatments often depend on the expression of specific antigens or proteins; however, the variability in antigen expression across tumor cells limits their ability to uniformly target all tumor cells. Consequently, incomplete tumor elimination may occur, allowing residual cancer cells to survive and develop immune evasion mechanisms, ultimately reducing therapeutic efficacy.^166^, ^173^, ^198^, ^199^, ^200^, ^201^

### 8.3 Instability and mRNA degradation:

Chemically synthesized exogenous mRNA molecules are inherently unstable and highly susceptible to degradation once introduced into the circulation. For effective delivery, mRNA must remain intact long enough to reach target sites, such as lung cancer cells, and exert its therapeutic effect.^78^ During synthesis, several stabilisation strategies are incorporated, including optimisation of the 5′ cap structure, inclusion of protective 5′ and 3′ untranslated regions (UTRs), extension of poly(A) tails, and nucleoside modifications.^19^, ^202^, ^203^ However, the TME of lung cancer is often characterised by altered metabolic and regulatory conditions, including dysregulated RNA-modifying enzymes, such as N6-methyladenosine (m^6^A) regulators. These enzymes can alter mRNA stability and translation efficiency and may also activate innate immune sensors, such as TLR3/7/8, RIG-I, and MDA5, leading to type I interferon responses that accelerate RNA decay.^200^, ^204^

### 8.4 Production, manufacturing, and regulatory limitations:

mRNA-based therapeutics face significant production, manufacturing, and regulatory challenges that highly affect preclinical development, clinical translation, and large-scale commercialisation.^205^ The design and production of mRNA therapeutics rely on in vitro transcription (IVT), a cell-free synthesis process that enables high-yield production of mRNA molecules.^206^ Rigorous characterization and purification are essential to prevent cellular degradation and reduce the instability of exogenous mRNA therapies.^206^ Manufacturers must produce highly purified mRNA, free from double-stranded and other RNA impurities, often relying on costly purification techniques, such as high-pressure liquid chromatography (HPLC), to improve product quality.^207^ In addition, regulatory frameworks for mRNA therapeutics are still evolving, requiring strict evaluation of safety, efficacy, quality control, and batch-to-batch consistency. Ensuring reproducibility and minimizing variability between batches remain major challenges, thereby increasing the complexity of manufacturing processes. Furthermore, mRNA therapeutics are considered combination products, consisting of both the active mRNA molecule and its delivery system, which adds a layer of regulatory complexity. Collectively, these barriers limit clinical scalability, restrict patient access, and raise concerns regarding the cost-affordability of mRNA-based therapies in lung cancer.^16^, ^19^, ^200^

### 8.5 Dosing, translational efficiency, and protein expression:

Many mRNA-based treatments for cancer are in the early phases of development, and critical parameters, such as optimal dose, administration frequency, treatment duration, and delivery routes, have not yet been defined.^84^ These factors must be thoroughly addressed before clinical translation and large-scale application. Like other cancer drugs, mRNA therapies are subjected to intensive studies to gain insights into pharmacokinetics (PK) and pharmacodynamics (PD) properties to better understand their distribution, stability, and biological activity.^208^ Determining the appropriate dose is particularly challenging, as excessive dosing may lead to off-target effects and strong inflammatory reactions due to activation of the innate immune system, whereas insufficient dosing may result in reduced therapeutic efficacy. Importantly, administered mRNA molecules do not directly correlate with the level of protein expression in target cells.^209^ Even when mRNA is successfully delivered, the translation rate can vary depending on factors, such as mRNA design, delivery vector characteristics, and the properties of both the healthy and TME.

A further pharmacokinetics challenge arises upon the entry of LNPs into the systemic circulation, where plasma proteins rapidly adsorb onto the surface of the nanoparticles, eventually forming a structure known as “protein corona”.^210^, ^211^ This formation alters the biodistribution of the LNPs, masks targeting features, and affects cellular uptake.^211^, ^212^ Furthermore, the corona structure may become enriched in complement proteins that promote immune-mediated clearance through the activation of the phagocytic system, thereby reducing circulation time and effectiveness. Moreover, the adsorbed protein may influence intracellular trafficking, potentially redirecting the LNPs toward lysosomal degradation pathways which in turn reduces the mRNA translation even when cellular uptake is achieved.^213^

In addition, repeated administration may be needed to support therapeutic effects, which can trigger an anti-LNP immune response and reduce treatment efficiency over time. Overall, achieving a balance between efficacy, safety, tissue specificity, and sustained protein expression is still a major challenge in the clinical development of mRNA-based therapies.^19^, ^173^, ^202^

## 9 Future directions

Recent advancements and adaptability of mRNA technology are transforming and expanding the concept of precision medicine for cancer treatment. Unlike conventional therapies, mRNA-based treatments enable the development of personalized treatments based on a patient's molecular and immunological tumor profiles. Current research efforts are focused on advancing next-generation sequencing (NGS) technologies, developing novel mRNA platforms, including self-amplifying mRNA (saRNA) and circular mRNA (circRNA), and integrating bioinformatics and AI-based tools to identify tumor-specific neoantigens for vaccine design.

Considerable progress is being made to improve delivery systems, which remains a major barrier to the clinical transition of mRNA therapeutics in lung cancer. Current strategies include the use of chemical modifications to enhance mRNA stability as well as the engineering of more robust nanoparticle formulations capable of withstanding physiological and mechanical stresses during pulmonary delivery. Targeting strategies are also evolving, with efforts directed toward incorporating ligand-based and dual-targeting approaches to enhance lung-specific delivery and overcome anatomical and biological barriers.

Future research should prioritize improving tissue-specific biodistribution, optimizing routes of administration, prolonging therapeutic antigen expression, reducing dose requirements, and minimising excessive immune activation. Addressing these challenges will be critical for improving long-term safety, therapeutic efficacy, and enable clinical applicability of mRNA-based therapies in lung cancer.

## 10 Conclusions

The molecular complexity of lung cancer, characterised by heterogeneity, genomic instability, and tumor plasticity, continues to limit the effectiveness of conventional therapies. For this reason, mRNA-based approaches have emerged as a promising paradigm shift toward more personalized and adaptive treatment strategies by enabling the development of advanced therapeutic modalities, including antigen-specific vaccines, immune response modulation, restoration of key tumor suppressor pathways, and gene editing. Their versatility enables their integration into combination plans designed to reshape the TME and enhance the patients' responsiveness. Despite these advances, mRNA-based therapies remain in early clinical stages, and current data remain insufficient to estabilish their long-term therapeutic efficacy and clinical impact. A major challenge is the lack of tissue specificity of current delivery systems, particularly the liver tropism associated with LNPs, which limits effective pulmonary tumor targeting. In addition, several immune-related challenges remain associated with mRNA theraputics, including unintended innate immune activation, variability in antigen presentation, and the immunosuppressive nature of the TME. Ongoing translational efforts are focusing on optimizing delivery systems, improving molecular stability, and ensuring the long-term safety of these therapies. Further research should prioritize the development of next-generation delivery systems with enhanced lung-targeting capability and improved biodistribution profiles. Moreover, mRNA therapy must be integrated with targeted therapies (e.g., EGFR and KRAS inhibitors) when designing the combination treatment strategies. In parallel, multi-functional mRNA strategies combining vaccines, cytokine-encoding mRNAs, and gene-editing platforms may act synergistically to enhance theraputic efficacy. Advances in computational modeling and systems-level approaches may further improve mRNA stability and design. In conclusion, mRNA-based approaches hold significant potential to transform lung cancer treatment, offering a versatile and powerful platform for future precision oncology applications.

## CRediT authorship contribution statement

**Iman Wehbe:** Writing – review & editing, Writing – original draft, Visualization, Validation, Data curation, Conceptualization. **Abdelaziz Tlili:** Writing – review & editing, Validation, Supervision.

## Declaration of competing interest

The authors declare that they have no known competing financial interests or personal relationships that could have appeared to influence the work reported in this paper.
